# PW02-015 - Eight years HPFS experience in a single UK centre

**DOI:** 10.1186/1546-0096-11-S1-A155

**Published:** 2013-11-08

**Authors:** DM Rowczenio, H Trojer, G Wang, PN Hawkins, HJ Lachmann, A Baginska, T Russell, R Al-Nackkash, A Bybee

**Affiliations:** 1 Medicine, University College London, London, UK

## Introduction

In 2004 we established a formal clinical service for patients with hereditary periodic fever syndromes (HPFS). Patients are either referred directly for clinical evaluation, or undergo initial genetic screening on receipt of blood or DNA sample and clinical details via our secure online request procedure: http://www.ucl.ac.uk/medicine/amyloidosis/nac/genetic_testing

## Objectives

To evaluate eight years experience of a dedicated fever clinic and associated laboratory service at the National Amyloidosis Centre.

## Methods

Between 2004 and 2009 the basic genetic screen encompassed FMF gene *MEFV* exons 2 and 10*;* TRAPS gene *TNFRSF1A* exons 2-6; MKD gene *MVK* exons 9 and 11 and CAPS gene *NLRP3/CIAS1* exon 3. When deemed appropriate, these tests were extended to additional exons on a case by case basis. From 2010 additional genes were added to our repertoire: *NOD2* (exons 2 and 4) associated with Crohn’s Disease and Blau Syndrome; *NLRP12* (exons 2 and 3) associated with familial cold autoinflammatory syndrome 2 and *IL36RN* (exons 2-5) associated with DITRA.

In each case the specific analyses were determined by the NAC physicians after clinical assessment or on the review of information provided by the external clinician.

## Results

Since January 2005, 3063 patients have undergone genetic screening at the NAC; 996 were assessed directly at the fever clinic (33%), and blood or DNA were received on a further 2067 (67%). *MEFV* was most frequently requested (75%), followed by *TNFRSF1A* (58%), *MVK* (42%) and *NLRP3* (29%), other genes accounted for 6%.

Genetic variants were identified in 1048 patients (34%): 627 (60%) had an amino acid variation in *MEFV* (56% had a single variant including 34% who had E148Q, 33% were compound heterozygotes and 11% homozygotes). *TNFRSF1A* variants were found in 133 cases (13%)*; MVK* in 74 (7%) (in 58% we were unable to detect a second variant despite screening of all exons); *NLRP3* in 103 (16%) and *NOD2* in 46 (4%). In 19 patients we identified genetic aberrations in more that one HPFS gene. 28 novel variants were discovered: 9 in *NLRP3*; 6 in *TNFRSF1A*; 7 in *MVK*; 5 in *MEFV and* 1 in *NOD.*

## Conclusion

Since creation of the HPFS clinical service in 2004, demand for genetic testing has grown substantially, including a 125% increase in referrals during the past year. In many cases two or more HPFS genes were screened. We found 1234 genetic variants in 1048 screened cases, of which nearly 40% accounted for low penetrance sequence variants of undetermined significance including *MEFV* E148Q, *TNFRSF1A* R92Q and P46L, *MVK* S52N and *NLRP3* V198M and Q703K. Interestingly we found 19 patients with variations in more than one HPFS gene. In 16 of these we were able to make a clinical diagnosis: 7 had CAPS; 5 MKD; 3 TRAPS and 1 FMF; the remaining 3 had atypical autoinflammatory phenotypes.

An overlap in clinical features between different HPFS highlights the importance of genetic testing in providing accurate diagnosis, leading to appropriate treatment and improvement of quality of life.

## Disclosure of interest

None declared.

